# A Naturalistic Approach to the Hard Problem of Consciousness

**DOI:** 10.3389/fnsys.2019.00058

**Published:** 2019-10-25

**Authors:** Wolf Singer

**Affiliations:** ^1^Max Planck Institute for Brain Research (MPI), Frankfurt am Main, Germany; ^2^Ernst Struengmann Institute for Neuroscience in Cooperation with the Max Planck Society (ESI), Frankfurt am Main, Germany; ^3^Frankfurt Institute for Advanced Studies (FIAS), Frankfurt am Main, Germany

**Keywords:** consciousness, dualism, social realities, qualia, emergence, self-model

## Abstract

Following a brief review of current efforts to identify the neuronal correlates of conscious processing (NCCP) an attempt is made to bridge the gap between the material neuronal processes and the immaterial dimensions of subjective experience. It is argued that this “hard problem” of consciousness research cannot be solved by only considering the neuronal underpinnings of cognition. The proposal is that the hard problem can be treated within a naturalistic framework if one considers not only the biological but also the socio-cultural dimensions of evolution. The argument is based on the following premises: perceptions are the result of a constructivist process that depends on priors. This applies both for perceptions of the outer world and the perception of oneself. Social interactions between agents endowed with the cognitive abilities of humans generated immaterial realities, addressed as social or cultural realities. This novel class of realities assumed the role of priors for the perception of oneself and the embedding world. A natural consequence of these extended perceptions is a dualist classification of observables into material and immaterial phenomena nurturing the concept of ontological substance dualism. It is argued that perceptions shaped by socio-cultural priors lead to the construction of a self-model that has both a material and an immaterial dimension. As priors are implicit and not amenable to conscious recollection the perceived immaterial dimension is experienced as veridical and not derivable from material processes—which is the hallmark of the hard problem. These considerations let the hard problem appear as the result of cognitive constructs that are amenable to naturalistic explanations in an evolutionary framework.

## Introduction

Attempts to provide naturalistic explanations for the phenomenon of consciousness are confronted with at least three major difficulties. The first arises from the fact that the explanandum is not well defined. The second results from the still rudimentary understanding of neuronal processes underlying higher cognitive functions. And the third is related to the “hard problem” of consciousness research (for review, see Dennett, 2018), the intuition that even if we had a comprehensive account of the neuronal correlates of consciousness (NCCP) we would still be unable to explain how the first-person experiences of the results of conscious processing, the qualia, emerge from the neuronal interactions described from a third-person perspective.

In this essay I shall briefly address the first two problems by reviewing recent developments in the search for the NCCP and then propose a strategy to soften the hard problem. The proposal is that the immaterial nature of the qualia can perhaps be accounted for within a naturalistic framework if one considers that not only the perception of the world around us but also the perception of ourselves is the result of a constructivist process that depends on priors. The core assumption is that the priors for our self-model are provided by the socio-cultural environments, the immaterial social realities, that humans have created once cultural and biological dimensions became interactive in evolution. Some of the experimental results pertinent to the NCCP have been reviewed by this author in previous articles and it is, therefore, likely that fragments of formulations are repeated here.

## An Ill-Defined Explanandum

The terms consciousness, conscious and consciously are associated with many different connotations and therefore discussions on neuronal correlates of consciousness are carried out on widely differing levels. In the most straight forward sense, the adjective conscious is used to simply designate brain states enabling subjects to be aware of their actions and their environment. In this case, consciousness is contrasted with sleep or coma. Defined in this way consciousness is a phenomenon that humans share with many different species of widely differing complexity. In another context, consciousness refers to a processing mode that is associated with verbal reportability of perceived stimuli or with storage of perceived items in working and/or declarative memory. The contrasting processing mode is subconscious or non-conscious processing in which stimuli are readily analyzed by the brain and can even control responses without the subject being aware of having been engaged in a cognitive operation. As far as can be assessed from a third-person perspective, all mammals and probably all vertebrates seem to be able to exploit these different processing modes. Although verbal reportability cannot be used as criterion, the brains of these animals possess all the mechanisms qualifying for conscious processing such as the control of attention, the storage of attended contents in working and episodic memory, the evaluation of context, the intentional selection of appropriate behavioral reactions and the ability to purposefully navigate in complex foraging grounds. In yet another reference frame consciousness is equated with a form of meta-awareness and denotes a condition, in which subjects are aware of their body scheme, of having emotions and memories or being in a particular state or having performed an action. This connotation of consciousness requires a form of self-awareness and is often assessed with the mirror test. A test that human babies pass beyond the age of two and certain animals such as craws, monkeys and apes. Yet another connotation of consciousness is the ability to generate a theory of mind, the ability to imagine what is in the mind of the respective other. For long this has been considered as a specific human ability but there is now robust evidence that at least birds, dogs and monkeys have this capacity as well. Finally, there are higher forms of meta-awareness that are thought to exist only in humans and are associated with self-reflection and self-control, leading to the attribution of responsibility, morality and free will. Here the implicit assumption is that only contents that enter consciousness are amenable to rational deliberations and decisions. And last but not least humans are conscious of being conscious, which might be considered the highest form of meta-awareness. Experts in contemplative practices claim in addition, that such states of meta-consciousness can be devoid of content, the conscious state being the only object of the “inner eye.” In view of these very diverse meanings attributed to the term consciousness it is natural that the strategies applied in the search for the NCCP are heterogeneous and address only selected aspects of consciousness.

For the sake of brevity, I shall not review work aimed at the identification of mechanisms controlling brain states that permit or prevent the manifestation of conscious behavior. In this field of research, there is broad consensus that a critical level of network excitability is required to enable conscious processing and that these states are characterized by dynamics with specific electrographic signatures. The mechanisms controlling these states are closely related with ascending modulatory systems that regulate the sleep-waking cycle and global levels of excitability. Rather I shall concentrate on the discussion of neuronal processes that distinguish conscious from non-conscious processing in the awake brain.

## Conscious and Subconscious Processing

At any one moment, subjects are only aware of a small fraction of their cognitive and executive operations. Still, signals that subjects are not aware of can be processed in considerable depth and impact behavior (Dehaene et al., 1998). Thus, there must be gating mechanisms that determine which signals are processed consciously, which are processed and control behavior but remain unconscious and which are not processed at all.

In animal experiments, one of the methodological problems is to assess from a third-person perspective whether a content had been processed consciously or subconsciously. It is commonly held that conscious processing is distinguished from subconscious processing by the ability of subjects to be *aware* of the consciously processed content and to report this fact. These contents can be percepts, thoughts, decisions, intentions and actions or, in the case of contemplative practices, the awareness of pure presence. Thus, the experimenter has to rely on reports from the subjects’ first-person perspective. In human subjects, this problem can be mitigated by requesting verbal reports or by instructing subjects to grade their operant responses according to the experienced degree of awareness. This allows one to overcome the ambiguity introduced by the fact that in most forced-choice paradigms subjects give correct responses well above chance even if they have not been aware of having perceived the stimuli. Experiments on blind sight and investigations of split-brain patients impressively document this fact (see below). As in animals only operant responses can be obtained, it is arguable whether distinctions between conscious and unconscious processing can be made in the same way as in experiments with human subjects. The mere difficulty or complexity of an accomplished task is only a weak indicator for the involvement of conscious processes because subconscious computations can also rely on highly sophisticated heuristics, semantic interpretations and logical deductions.

In depth exploration of neuronal mechanisms requires assessment of neuronal responses with high temporal and spatial resolution, and this is also the case for the investigation of the NCCP. With the exception of utilizing data from patients implanted with intracranial electrodes for diagnostic reasons, such high resolution data can only be obtained in animal studies. Yet, these approaches are hampered by the ambiguities associated with operant responses. Thus, results from animal studies are usually interpreted on the basis of the assumption that the distinction between conscious and subconscious processing also holds at least for higher vertebrates and mammals. The reason is the remarkable cross species similarity of brain organization. However, as pointed out by the philosopher Nagel (1974) we cannot know whether animals are aware of stimuli and responses in the same way as human subjects.

For this very reason, most studies on the NCCP are performed with non-invasive measurements in human subjects. However, because of the limited spatial and/or temporal resolution of these techniques, mechanistic interpretations often have to rely on analogies with neuronal processes supporting cognitive functions in animals that are only indirectly related to consciousness.

One important mechanism gating the access of information to conscious processing, most likely shared with other mammals, is attention. As suggested by the phenomena of change blindness (Simons and Chabris, 1999), sensory neglect (Doricchi et al., 2008), attentional blink (Fu and Rutishauser, 2018), and certain masking paradigms, non-attended stimuli usually fail to be processed consciously and escape the subjects’ awareness. Whether these limitations are due to the inability to attend to large numbers of items simultaneously or whether they result from the restricted capacity of working memory or the workspace of consciousness is subject to intense scientific investigation. In any case, capacity constraints limit the number of items simultaneously amenable to conscious processing. Which contents eventually reach the level of conscious awareness depends either on external cues that attract attention or on internal selection processes that direct attention either to external inputs or to material stored in memory. Most of the time subjects are not aware of performing such selections which gives rise to the impression that what surfaces in consciousness is all there is. Interestingly even conscious, intentional search for a content safely stored in declarative memory may fail to move that content into the workspace of consciousness. It is often a persistent non-conscious search process that suddenly lifts the searched items into the workspace of consciousness. This indicates that access to consciousness is only partly under the control of the conscious agent itself.

## The Classical Experimental Paradigms: Results and Limitations

The most frequently applied strategy for the identification of the NCCP consists of creating conditions in which physically identical stimuli are processed consciously only in a fraction of trials and then to subtract neuronal activation patterns associated with non-conscious processing from those generated during conscious processing. The assumption is that the remaining activation patterns are characteristic for conscious processing. However, as discussed by Aru et al. (2012b), this approach is fraught with numerous ambiguities.

The subtractive procedure uncovers not only the hypothetical NCCP proper. It reveals also the various processes that gate access to consciousness and the many processes that follow once subjects became aware of a stimulus. Among the latter are the transfer of information into working and episodic memory, the covert preparation of motor responses and in case of human subjects the covert verbalization of perceived contents. Because of dense reciprocal coupling between brain areas and the prevalence of parallel processing, segregation of these confounding factors is notoriously difficult with non-invasive recording techniques. Thus, there is the caveat that data obtained with this method may reflect not only the NCCP proper but also prerequisites for and consequences of conscious processing.

Here is an example: patients implanted with subdural electrodes over the visual cortex performed a recognition task in which the visibility of faces was manipulated either by increasing sensory evidence or providing a-priory knowledge (Aru et al., 2012a). The reasoning was that activity patterns specific for the NCCP proper should be the same irrespective of whether stimuli were consciously perceived because of enhanced sensory evidence or because of top-down facilitation. In trials in which conscious perception was caused by increasing sensory evidence there was indeed a category specific enhancement of gamma oscillations in the fusiform face area, suggesting that this increase in synchrony of neuronal responses had to do with conscious perception. However, this increase was lacking when sensory evidence was kept constant and visibility enhanced by prior knowledge. This suggested the conclusion “that the differential activation of specific areas of the visual cortex is a necessary but not a sufficient condition for conscious processing” (Aru et al., 2012a).

Other frequently applied paradigms for the identification of the NCCP manipulate the context of stimulus presentation with the aim to abolish conscious perception (reportability) of subsets of identical stimuli. This is achieved by exploiting interocular rivalry, masking paradigms, priming and variations of signal to noise ratios. So far, these approaches have yielded inconclusive results. Exploiting binocular rivalry, Leopold and Logothetis (1996) found that responses to perceived and non-perceived stimuli differ only at higher processing stages of the ventral stream. They concluded that activation of neurons in the inferotemporal cortex, one of the highest levels in the visual processing hierarchy, qualified as NCCP. However, Fries et al. (1997) discovered in the primary visual cortex of cats that responses of cells to perceived stimuli differed from those to non-perceived stimuli because of increased synchronization of oscillatory responses in the gamma frequency band (Fries et al., 1997). This suggests that at this early stage of processing increased synchronization rather than increased discharge rate allows these responses to compete successfully with the conflicting inputs from the suppressed eye in the respective upstream target areas. These results agree with psychophysical and non-invasive tract tracing studies in human subjects, which indicate that interocular rivalry and hence the gating of access to consciousness involves already mechanisms in primary visual cortex (Genç et al., 2011). Finally, combining rivalry experiments with fMRI measurements in human subjects revealed reduced responses to the respective suppressed eye as early as in the lateral geniculate body, the thalamic relay for retinal signals (Haynes et al., 2005).

Thus, conscious perception seems to involve also early stages of sensory processing. This view is supported by the evidence that imagery, the visualization of imagined contents, is associated with increased BOLD activity in primary visual cortex and that lesions of primary visual cortex lead to blindsight. Patients with such lesions can still use visual information for orienting responses and avoidance of obstacles but they cannot consciously perceive visual stimuli (for review, see Goebel et al., 2001). These results indicate that appropriate activation of primary sensory areas of the cerebral cortex is a necessary prerequisite for the mediation of conscious perception but they do not allow the conclusion that this is a sufficient condition.

Another class of experiments makes use of clinical syndromes that go along with disturbances of conscious perception as is the case in patients with blind sight (Weiskrantz, 2004), neglect, agnosia, section of the commissures (split brain) or reduced states of consciousness (for discussion of some of these approaches see the other contributions of this volume).

Signals amenable to conscious processing can obviously also originate within the brain itself. Examples are signals associated with the recall of memories, imagery, decision making, planning, deliberating and reasoning. Thus, conscious experience appears to result from very versatile cognitive processes that can recruit neuronal activation patterns from many different sources and bind them together in a unified format.

Finally, indications for the substrate of the NCCP are derived from the evidence, that a plethora of signals from specialized receptor systems are excluded from conscious perception even though these signals are processed in great depth by the brain and exert strong control over behavior. Examples are enteroceptive signals that maintain metabolic homeostasis, pheromone signals controlling reproductive behavior and signals for the synchronization of circadian rhythms. Unlike the classical five senses, these signaling systems are not represented by devoted cortical areas, supporting the view that cortical structures are involved in the mediation of conscious experience.

## Two Non-exclusive Hypotheses: Anatomical Substrate vs. Dynamical State

Current theories about the nature of the NCCP can be grouped into two major, non-exclusive clusters. The first assumes that particular brain structures have to be engaged to permit conscious processing. The idea is that these structures subserve what is sometimes addressed as the “inner eye function.” In this case it is assumed that the contents of conscious experience are represented and bound together by a distinct structure onto which the various processing streams would converge. This structure would have to be positioned at the top of the processing hierarchy. The second group of theories assume that conscious and non-conscious processes could involve the same anatomical substrate but differ with respect to dynamic states reflecting the degree of integration of distributed processes. As candidates for such state variables have been proposed temporal coherence, synchrony, correlation length and dimensionality.

### Binding in the Spatial Domain

Identification of brain structures whose activation is crucial for conscious processing is problematic if the distinguishing criterion is reportability. In this case a considerable number of brain structures and networks would qualify: the entire dominant hemisphere in split-brain patients, the parietal cortex in case of neglect, multiple sensory areas in case of agnosia, and ultimately the language system itself or structures required to access the language system. Split-brain experiments illustrate this problem. Stimulus material presented to the sensory space contralateral to the non-dominant hemisphere is often not reportable even though patients readily process the respective information and generate adapted motor responses. Rather than taking this as evidence that conscious processing is tied to the dominant, speech-competent hemisphere one could argue that the disconnection simply prevents the dominant, speech competent hemisphere from reporting. Although this disconnection jeopardizes language-dependent post-processing steps such as rational deliberation it would seem strange to deny the otherwise intact and awake non-dominant hemisphere the ability to sustain consciousness. If one were to reach this conclusion, one would have to deny that animals are conscious which is clearly untenable. The proposal that there is a special work space for conscious processing, promoted by Baars (1997) and later by Dehaene et al. (2006) also makes assumptions on the involvement of specific structures, in this case the ensemble of reciprocally coupled neuronal groups located in the supragranular layers of the cerebral cortex. However, it is difficult to provide causal evidence for this hypothesis because inactivation of supragranular layers would also jeopardize all the other functions of the cerebral cortex. The intuitively plausible hypothesis that the contents of conscious experiences are represented and bound together in a distinct structure at the top of the processing hierarchy is thus not well supported by experimental evidence. As argued by Dennet (1992), a region with such universal “observer functions” would be theoretically implausible. Moreover, behavioral and brain imaging studies have shown that unconscious and conscious processing engage very much the same brain regions, including frontal and prefrontal cortex (Lau and Passingham, 2007; van Gaal et al., 2008). Which of the respective areas get recruited into functional networks depends more on the nature of the task than on the mode of processing.

### Binding in the Temporal Domain

Probably the first to propose that conscious and non-conscious processes could involve the same anatomical substrate but differ with respect to dynamic states reflecting the degree of integration of distributed processes was Sherrington (1906). He proposed that the unity of consciousness could be achieved by binding the contents of conscious experience in the temporal domain. In his book “The Integrative Action of the Nervous System” he stated: “Pure conjunction in time without necessarily cerebral conjunction in space lies at the root of the solution of the problem of the unity of mind.” He proposed that the unity of consciousness does not necessarily require anatomical convergence but could be achieved by convergence in time. The idea that temporal rather than spatial integration is a necessary prerequisite for conscious processing is at the basis of numerous recent theories and experimental evidence in favor of this notion keeps increasing.

Baars (1997) proposed that there is a special workspace for conscious processing and that subjects become aware of signals if these are sufficiently salient to ignite coordinated activity within this workspace. As mentioned above, Dehaene et al. (2006) and Gaillard et al. (2009) proposed the neuronal correlate of this workspace to be a widely distributed network of neurons located in the superficial layers of the cortical mantel. This network, so the assumption, would be “ignited” if a sufficient number of nodes were activated together.

Others have suggested that this “workspace” should be seen not so much as a sub-compartment of the cerebral cortex but as a special dynamic state of the brain that favors large scale *binding* of the results of widely distributed cortical computations (e.g., Varela et al., 2001; Melloni et al., 2007; Gaillard et al., 2009; Oizumi et al., 2014). According to the binding by synchrony hypothesis (Singer, 1993, 1999) it has been proposed that the respective dynamic state should be characterized by enhanced coherence of oscillatory activity in the beta or gamma frequency range. Using the subtraction method (see above) experimental evidence could be provided that processing of consciously perceived stimuli was indeed associated with better synchronization of large cortical networks in the beta and gamma frequency range than processing of identical stimuli that had failed to reach the threshold for awareness and remained invisible (Melloni and Rodriguez, 2007; Melloni et al., 2007; Gaillard et al., 2009; Melloni and Singer, 2010).

Contents that one is aware of are experienced as simultaneously present and related to each other. Thus, a mechanism is required that permits flexible and fast association of the ever-changing contents of conscious experience into a coherent whole. Dynamic binding by transient synchronization of widely distributed processes could in principle fulfill such a function.

More recently a related hypothesis, the “Information Integration Theory” has been formulated by Tononi (2004; see also Oizumi et al., 2014). This theory also posits that conscious processing is associated with particularly effective and global integration of information from different sources. Supportive evidence for this conjecture comes from several independent observations. First, dynamic states that favor conscious processing such as arousal and attention facilitate the propagation of excitatory perturbations over larger cortical distances, which is likely to enhance interactions between distributed processes (Massimini et al., 2005). Second, arousal and attention facilitate synchronous oscillations in the gamma and high beta frequency range (Herculano-Houzel et al., 1999; Fries et al., 2001; Lima et al., 2011). This oscillatory patterning of activity, in turn, facilitates long-range synchronization (Roelfsema et al., 1997; for review, see Singer, 1999) and thereby enhances communication between remote groups of neurons and the formation of large scale functional networks (Roelfsema et al., 1997). A mechanistic account for the “binding” function of synchronization has been formulated in the “communication by coherence” (CTC) hypothesis (Fries, 2005) that has in the meantime received experimental support (Womelsdorf et al., 2007; Bastos et al., 2015).

The reportability that is considered as such a critical feature of conscious processing could thus be a natural consequence of a highly integrated processing mode. In human subjects integration of widely distributed processes would automatically involve the language network because of its particularly strong interconnections with both sensory and executive systems. Thus, reportability would simply be a consequence of processing modes characterized by a particularly high degree of integration but not a necessary requirement for conscious processing. This is a further argument for the notion that animals, at least those with highly evolved brains such as vertebrates, can switch between conscious and unconscious processing modes. The circuitry of their brains and the ensuing dynamics can certainly sustain highly integrated processes.

## The Hard Problem

Even if we had a full account of the NCCP, of the neuronal mechanisms whose involvement distinguishes conscious from non- conscious processing, the “hard problem” in consciousness research would persist. We still would have no explanation for the phase transition from material neuronal processes, described from a third-person perspective, to the immaterial mental phenomena, that we experience from our first-person perspective. In the following I shall attempt to narrow this explanatory gap by attempting a naturalistic, evolutionary explanation for the fact that many humans experience themselves as having both a material and an immaterial mental or spiritual dimension. The core of the argument is that the gap can probably not be closed if one considers only the cognitive functions of individual brains but that in addition the phenomena emerging from *social* *interactions* have to be taken into account. This extension requires joint consideration of evidence and analyses from philosophy, cognitive neuroscience, cultural anthropology, developmental psychology and social science. The arguments are based on the assumptions that: (i) perceptions are the result of a constructivist process that depends on priors; (ii) social interactions lead to the emergence of a novel class of realities, the immaterial social and cultural realities; (iii) these cultural realities assume the role of priors for perception; and (iv) the construction of the self-model is based on experiences that are shaped by cultural priors.

## The Constructivist Nature of Perceptions

Abundant psychophysical and neuroscientific evidence indicates, that our perceptions are the result of complex computations in which sparse sensory evidence is interpreted on the basis of a huge amount of prior information about the world (for review, see Spratling, 2017). This information is contained in the functional architecture of the brain. Part of this knowledge has been acquired through evolutionary selection and is stored in the genes. This inherited knowledge is then complemented by experience-dependent development and adult learning. The knowledge acquired during evolution and early development is implicit, i.e., the perceiving agent is not aware of its existence although it plays an essential role in determining the agent’s perception. Hence, the validity of the perceptions shaped by these implicit priors cannot be questioned. The perceiving agent cannot but take as real what she/he perceives. By contrast, priors acquired by learning later in life are to some extent amenable to conscious recollection and perceptions shaped by these explicit priors can be challenged by reasoning (conscious deliberations). A core assumption of the present proposal is, that all perceptions, regardless as to whether they result from stimuli in the external world or from introspection depend on priors.

## The Emergence of Social Realities

Biological evolution has brought forth organisms with increasingly refined cognitive functions: the ability to develop a theory of mind by taking the perspective of the respective other, to generate abstract descriptions by recognizing similarities among seemingly different appearances through poly-sensory integration, to represent these abstractions in symbolic form and to eventually communicate the results of these cognitive functions. Recent studies suggest that evolved animals such as birds and mammals possess some of these abilities in various combinations and often in rudimentary forms. Humans excel because they possess all of these functions and in addition have developed language which allows them to communicate the results of their cognitive operations in a highly abstract and symbolic way. Once agents endowed with this unique combination of cognitive abilities began to cooperate and to communicate with each other, they began to create a new class of phenomena that the philosopher John Searle addressed as “social realities.” These are immaterial realities that evolution brought into this world once development of sophisticated cognitive abilities allowed organisms to engage in social interactions. The rules governing the coexistence of social animals can be regarded as rudimentary forms of such social realities. However, in human societies these immaterial realities are no longer just implicit forces that coordinate cooperativity but assumed the status of realities that became consciously perceivable as integral part of the world. Examples of social realities created by human societies are empathy, fairness, greed, love, devotion, shame, norms, vows, commitments, social status, values, belief systems, laws, regulations and moral imperatives. These are realities that cannot emerge from the cognitive abilities of individual brains alone but require for their creation the interaction of at least two cognitive agents. They are immaterial, mental constructs but they are real in the sense that they can readily be perceived and strongly influence behavior. Believes in the reality of these immaterial constructs erect cathedrals and motivate suicidal behavior.

How then could such immaterial realities have emerged? Here is a likely scenario. A group of cave dwellers sits around a fireplace and shares food. Sooner or later members of this group will discover that there are certain subjects who are more generous, or greedier than others. If these observations are shared by a sufficient number of group members, generosity and greed will eventually acquire the status of perceivable realities and then can be symbolically represented by a common description. This scenario suggests as necessary prerequisites for the emergence of novel, immaterial realities: the collective experience of intangible, immaterial phenomena, the mutual affirmation of the reality/existence of these phenomena through shared attention and experience, the naming of these phenomena and finally the representation of these immaterial realities in rituals and artistic creations. These artistic activities retranslate the immaterial realities into concrete symbols that are then perceivable with the classical five senses.

## Social Realities as Priors of Perception

The second core assumption is that these immaterial realities assume the function of priors for perception in very much the same way as all the other inherited and acquired a priory assumptions about the world and ourselves. As a consequence of this cognitive embedding in a world in which tangible and immaterial realities coexist humans are bound to perceive reality as consisting of two classes of phenomena, objects perceivable with one of the five senses and immaterial phenomena that cannot be directly perceived. Priors of perception such as e.g., the Gestaltrules are usually implicit, i.e., subjects are not aware of the priors that shape their perceptions. Hence, humans—and animals as well—are bound to take what they perceive as evident and real. This is likely the case also for the perceptions shaped by cultural priors. As a consequence, individuals perceive cultural realities as equally evident and concrete as objects of the material world. What is perceived is taken for granted and experienced as true—and since cultural priors are shared by the members of communities there is usually a broad consensus about the reality of the perceived. A natural consequence of this shared perception of a dichotomous reality is the construction of an ontological substance dualism with its many different, culture-specific flavors.

## Substance Dualism and the Self-Model

The third core assumption of the attempt to soften the hard problem of consciousness research is that the self-perception of the conscious Self with all its immaterial connotations is a consequence of perceptions shaped by priors provided by social realities. Humans experience/perceive themselves as autonomous, cognitive and intentional agents and observations of the actions of the respective other do not contradict but confirm this perception. Humans are aware of being able to perceive, to reason, to decide and to act and they can share through language the contents of this meta-awareness and through comparison and observation of the respective other assure themselves of the reality of these immaterial properties of the conscious Self. However, humans have neither access to the priors that shape the perception of an immaterial Self nor do they have access to the material neuronal processes that underlie their cognitive and executive functions. All that humans can experience are the consequences of their brains’ actions and because they have been familiarized with the existence of immaterial realities they naturally postulate as cause of their actions an invisible, seemingly immaterial agent that is not constrained by the laws of nature and whom they equate with the conscious, intentional and responsible Self.

Thus, very much in the same way as proposed above for the construction of social realities, humans have shared their experiences on the existence of an immaterial agent, came to a similar conclusion, invented names for the various manifestations of this agent and assigned it to the realm of the intangible immaterial realities. Once this concept was commonly shared it likely assumed the status of a prior that henceforth shaped not only the perception of others but also the perception of one-self. In that way, it could have become seamlessly integrated in the self-model. This, in turn, could account for the fact that humans perceive themselves as existing both in a material and in an immaterial dimension. As perceptions based on implicit priors cannot be questioned and are experienced as true, human subjects are bound to take their dual nature, their existence in both a material and a spiritual domain for granted. Consequently all the properties of this immaterial agent, its feelings, intentions, beliefs, wishes and the contents of consciousness are attributed to the immaterial domain—in perfect agreement with the traditional view of ontological substance dualism.

The perception of a dualist reality is further reinforced by education, religions and shared belief systems. These normative systems emphasize the autonomy and independence of an immaterial agent that is unconstrained by the material, biological domain. They instrumentalize cultural priors in order to establish a self-model that permits to experience freedom and hence responsibility.

However, problems arose for this self-model once the natural sciences, in particular the neurosciences, set out to study the biological underpinnings of behavior and mental phenomena. One of these problems is the postulate of mental causation. If one adheres to ontological substance dualism, if one believes in the existence of an immaterial agent, the Self, that is independent of the neuronal processes in the brain and endowed with consciousness, intentions and free will, one needs to assume that this immaterial agent interacts with the brain so that the brain translates intentions and decisions into action. Such a scenario violates the known laws of nature, in particular, the law for the conservation of energy, because interactions with a material substrate require the exchange of energy. Per definition, however, the immaterial domain should be devoid of energy, otherwise it would again be part of the physical, the material domain.

The other, closely related problem is the “hard problem”, the explanatory gap between neuronal processes and the qualia of our experiences. The first problem vanishes if one is prepared to accept the overwhelming neurobiological and neuropsychological evidence that mental phenomena are the consequence and not the cause of neuronal interactions. The second problem is at least alleviated if one accepts: (i) that our perceptions are the result of a constructivist process that depends on priors; (ii) that this applies not only for perceptions of the outer world but also for perceptions based on introspection; and (iii) that social realities assume the role of priors for self-perception.

## The Emergence of New Qualities From Interactions in Complex Systems

**Figure 1:**
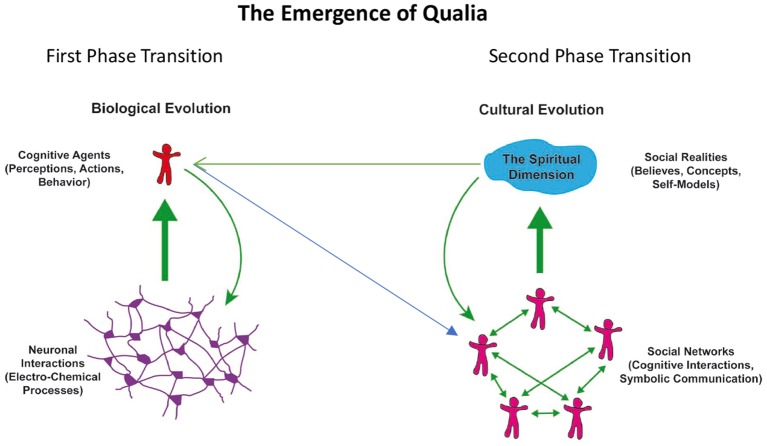
Schematic representation of phase transitions in the evolution of complex systems leading to the emergence (thick green arrows) of new qualities: interactions in neuronal networks (left) lead to cognitive and executive functions of autonomous agents. These agents form again networks (blue arrow) and interactions among these agents lead to the emergence of social realities (right). The new qualities act upon and alter the organization of the respective underlying substrates (green arrows).

From an evolutionary perspective, the awareness/experience of a mental or spiritual dimension and its integration in our self-model can be understood as a sequence of evolutionary phase transitions that characterize complex, self-organizing systems. For illustration of this concept see Figure 1.

The first phase transition is the emergence of cognitive functions from complex interactions in neuronal networks. The second phase transition is the emergence of social realities from the complex interactions in networks of cognitive agents. In both cases the emergent phenomena, the cognitive functions generated by neural networks and the social realities generated by social networks, transcend the properties of the components of the respective networks. The emergent phenomena cannot be understood by only considering the properties of the respective network nodes and they cannot be described with the terms used for the description of the nodes. Hence different language systems had to be developed to capture the properties of the emergent phenomena. There is a language for the description of neuronal processes, another one for the characterization of the emergent cognitive and executive functions, yet another for the description of cognitive agents in their role as nodes in social networks and finally there is a language to capture the emergent social realities. These language systems are each represented by different scientific disciplines, the second and third straddling the border between the natural sciences and the humanities and the fourth being entirely a domain of the humanities. Past attempts to relate consciousness to neuronal processes and to narrow the explanatory gap between the material neuronal processes and the qualia of subjective experience have considered only the first phase transition and by and large neglected the second—which is the likely reason why the gap is perceived as too large to be closed.

The arguments exposed in this contribution suggest that the attempts to identify the underpinnings of consciousness must not be confined to the analysis of the neuronal functions of individual brains but must include the domain of socio-cultural phenomena that are traditionally dealt with by the humanities. The present approach is partly based on assumptions whose validation is beyond my competence. These concern, in particular, the emergence of social realities, the function of social realities as priors of perception, the influence of these priors on self-perception and the constructivist nature of the processes that lead to the self-model. Some of these assumptions may have been verified already by empirical evidence in studies on cultural evolution, evolutionary anthropology and developmental psychology but in principle, they should all be amenable to empirical testing. Thus, research on the ill-defined explanandum “consciousness” seems ideally suited to bridge the still wide gap between the natural sciences and the humanities. If successful, such a comprehensive research agenda might be able to eventually settle the epistemic disputes on the nature of consciousness, on the problem of mental causation and on the relation between mind and matter. As I have tried to show, this synthesis should be realizable within a naturalistic, evolutionary framework that is based on empirical evidence. However, it requires joint consideration of phenomena that emerged from phase transitions in a continuous evolutionary process that comprises both biological *and* cultural evolution. Although this approach is incompatible with ontological dualism and qualifies the spiritual dimension as a cognitive construct it leaves sufficient space for the precious immaterial entities that are constitutive for human identity.

## Author Contributions

The author confirms being the sole contributor of this work and approved it for publication.

## Conflict of Interest

The author declares that the research was conducted in the absence of any commercial or financial relationships that could be construed as a potential conflict of interest.
